# Workplace wellbeing in community pharmacy practice: A cross-sectional study in Can Tho, Vietnam

**DOI:** 10.3934/publichealth.2024013

**Published:** 2024-03-11

**Authors:** Van De Tran, Trung Tin Pham, Trung Hieu Le, Thanh Thao Nguyen Thi, Minh Trung Nguyen, Duong Phuc Phan, Thi Bich Thuy Bui, Minh Cuong Nguyen, Rebecca Susan Dewey, Nguyet Tu Tran

**Affiliations:** 1 Department of Health Organization and Management, Can Tho University of Medicine and Pharmacy, 179 Nguyen Van Cu Street, Can Tho 900000, Vietnam; 2 Department of Epidemiology, Can Tho University of Medicine and Pharmacy, 179 Nguyen Van Cu Street, Can Tho 900000, Vietnam; 3 Department of Nutrition and Food Safety, Can Tho University of Medicine and Pharmacy, 179 Nguyen Van Cu Street, Can Tho 900000, Vietnam; 4 Administration Office, Faculty of Public Health, Can Tho University of Medicine and Pharmacy, 179 Nguyen Van Cu Street, Can Tho 900000, Vietnam; 5 Faculty of Pharmacy, Nam Can Tho University, 168 Nguyen Van Cu Street, Can Tho 900000, Vietnam; 6 Sir Peter Mansfield Imaging Centre, School of Physics and Astronomy, University of Nottingham, Nottingham NG7 2RD, United Kingdom; 7 Department of Environmental Health, Can Tho University of Medicine and Pharmacy, 179 Nguyen Van Cu Street, Can Tho 900000, Vietnam

**Keywords:** workplace wellbeing, community pharmacy, factor analysis, Vietnam

## Abstract

**Background:**

Among pharmacy workers, low workplace wellbeing can lead to reduced effectiveness. However, to date, studies on this issue are limited within the community pharmacy setting in Vietnam.

**Objectives:**

This study was conducted to identify the component aspects of workplace wellbeing and their associations with demographic characteristics.

**Methods:**

The cross-sectional descriptive study was conducted in Can Tho, Vietnam. Self-administered questionnaires were hand-delivered to all pharmacy workers working at selected community pharmacies. The workplace wellbeing scale comprised 18 items.

**Results:**

In total, 382 pharmacy workers participated in this study. Factor analysis revealed three fundamental aspects to workplace wellbeing: Factor 1 – perceived self-worth and job satisfaction, Factor 2 – positive emotions with work, and Factor 3 – negative emotions with work. Factor 1 showed a positive correlation with Factor 2, with a correlation coefficient (ρ) of 0.509, while both Factor 1 (ρ = −0.399) and Factor 2 (ρ = −0.416) demonstrated negative correlations with Factor 3. Higher income was associated with higher positive emotions with work (*P* = 0.008), higher perceived self-worth and job satisfaction (*P* = 0.013), and lower negative emotions with work (*P* < 0.001).

**Conclusion:**

Workplace wellbeing of pharmacy workers in their professional environments was associated with financial aspects. These findings suggest that policies aimed at improving income for pharmacy workers could bring benefits to enhancing job satisfaction and workplace wellbeing.

## 1. Introduction

Wellbeing is a crucial metric across individuals and societal domains, as a high level of wellbeing indicates a perception of success [1]. Wellbeing in turn affects several other domains in life, contributing to maintaining both physical and mental health, job success, life satisfaction, and the economic development of a nation [2],[3]. There is evidence suggesting that individuals with a high wellbeing exhibit higher workplace productivity, increased effectiveness at learning, increased creativity, improved social behaviors, and positive interpersonal relationships.

Wellbeing is a multidimensional construct that can be defined in a number of ways [1]. One common definition includes cognitive (life satisfaction) and affective (high positive affect, low negative affect) dimensions [4]. When applied to the workplace, wellbeing encompasses both positive and negative workplace influences and job satisfaction [5]. Workplace wellbeing is recognized as a fundamental factor in the success of an organization, leading to desirable outcomes such as higher employee retention rates and enhanced employee performance.

In the pharmaceutical industry, community pharmacies play a vital role in healthcare delivery and disease prevention [6],[7]. Community pharmacists are accessible healthcare professionals who are primarily responsible for ensuring the quality of and dispensing medications and ensuring their proper use by dispensing them alongside appropriate information [8]. Community pharmacists are important contributors to public health efforts, including disease prevention, immunization, opioid management, smoking cessation, and chronic disease management [9]. Community pharmacists also fulfill core responsibilities such as providing information about medication and counseling to patients, continuously updating their own professional knowledge, and ensuring medication safety through organization, storage, preparation, and dispensing [10].

Nowadays, there is a shift within the pharmacy profession toward patient-centered care, resulting in continuous changes in the role of the pharmacist. Community pharmacists must actively update their knowledge, clinical skills, and communication strategies to maintain their ability to interact with patients and other healthcare professionals [11]. The study by Nsengimana et al. (2022) revealed that 98.7% of community pharmacy professionals responded, stating that health promotion is part of their responsibility, and they are willing to provide health promotion services [11]. Additionally, the COVID-19 pandemic has added significant professional pressure on community pharmacists [12]. A study by Etezad et al. (2023) found that 85% of community pharmacy professionals reported that their mental health had been impacted since the COVID-19 pandemic [12]. Globally, pharmacists work under challenging conditions, facing numerous obstacles that can lead to low job satisfaction, decreased wellbeing, high burnout, and many people choosing to leave the profession [13],[14]. The instability created by high staff turnover in turn significantly disrupts the working environment of pharmacists and therefore impacts the quality of community healthcare delivered [13].

A study conducted in the United States revealed that 75% of community pharmacists experience burnout due to job demands and working conditions [15]. In Vietnam, the role of community pharmacists in improving community health has been established, similar to as in other parts of the world. As of 2022, there were 44,600 drug stores in Vietnam, across 1,600 pharmacy chains [16]. The density of drug outlets in Vietnam per 100,000 people is four times higher than the global average [17]. Over 80% of the population seek assistance from a community pharmacy as their first course of action when dealing with a health concern [10],[18].

Low wellbeing among community pharmacists can lead to decreased workplace effectiveness [19]. Therefore, researching the levels of wellbeing among retail pharmacy staff and identifying factors influencing their wellbeing is crucial for implementing the interventions necessary to improve workplace wellbeing. However, to the best of our knowledge, this concept is relatively new in the setting of community pharmacy in Vietnam, and research on this topic is limited. Thus, we aim to explore differences in the aspects of workplace wellbeing among community pharmacy professionals. Our results may lead the field toward suggestions, recommendations, and solutions for improving the wellbeing of pharmacists.

## 2. Materials and methods

### 2.1. Study setting

This cross-sectional descriptive study was conducted in community pharmacies across Can Tho City, Vietnam. Can Tho City is centrally located in the Mekong Delta region and consists of five urban districts and four rural districts. Urban districts were selected for this study due to their higher numbers of community pharmacies. The following four urban districts were randomly selected for this study: Ninh Kieu, Binh Thuy, Cai Rang, and Thot Not. The survey was carried out between April and May, 2023.

### 2.2. Study sample

A list of pharmacies across the four selected districts was compiled, and the address of each pharmacy was identified using Google Maps. From this list, individual pharmacies were selected for inclusion in the study using convenience sampling based on criteria such as the pharmacy's location and proximity to the central areas of each district. Two final-year pharmacy students were trained to directly approach the selected pharmacies and distribute printed questionnaires to the participants, collecting them immediately after completion. Each data collector was assigned two districts. All staff working at the selected pharmacies present at the time of the survey were invited to participate. Staff who were not present during the survey period were excluded from this study. There were 243 approached and agreed pharmacies for this study. A total of 400 pharmacy workers agreed to participate in the research. Correspondingly, 400 questionnaires were returned, of which 18 had missing values. Thus, the final sample included responses from 382 pharmacy workers in Can Tho, Vietnam.

### 2.3. Survey tool

A self-administered questionnaire was developed based on previous studies. The questionnaire comprised two sections, covering socio-demographic information and an assessment of workplace wellbeing.

In this study, multiple aspects of workplace wellbeing were measured, including levels of positive and negative affect experienced in the workplace and job satisfaction [5]. Building on Seligman's (2011) perspective on wellbeing, it is argued that experiencing wellbeing at work requires not only job satisfaction but also the perception of a sense of meaning to one's work [20]. Therefore, the concept of workplace wellbeing was further expanded to include the dimension of perceiving a sense of value in one's job. The wellbeing assessment scale consisted of 18 items, divided into four dimensions: negative emotion with work (4 items), positive emotion with work (4 items), job satisfaction (4 items), and perceiving a sense of value in one's job (referred to in this study as ‘self-worth’ for brevity) (6 items). Participants responded to each item using a 5-point Likert scale, ranging from 1 (strongly disagree) to 5 (strongly agree). This Vietnamese version of the wellbeing assessment scale was developed and validated in a previous study by Do and Phan (2019) [21]. However, as the scale was initially designed for the general working population, its validity was reassessed to ensure its appropriateness for healthcare professionals, specifically pharmacy workers, in our study.

### 2.4. Data analysis

The collected data were analyzed using SPSS Statistics 22.0 software. Questionnaires with any missing values or incomplete data will be excluded from the analysis of this study. Descriptive statistics, such as frequency and percentage, were used to describe categorical variables. In factor analysis, extraction method with Principal Component Analysis and Varimax rotation method was employed to identify the underlying factors within the wellbeing questionnaire. The factor analysis had to meet the following criteria: (i) Kaiser-Meyer-Olkin (KMO) measure of sampling adequacy ≥0.5 and a significant result (p-value) from the Bartlett's test of sphericity <0.05; (ii) minimum total extracted variance of 50%; (iii) eigenvalues of retained factors greater than 1; (iv) factor loadings of variables above 0.4; and (v) at least 3 items loading onto a single factor, and corrected item-total correlation >0.3. Factor scores were calculated as the sum of item scores within each factor, with higher scores indicating greater agreement with the aspects of job-related wellbeing. The differences in factor scores among groups of pharmacy workers classified by socio-demographic and professional characteristics were evaluated using the Mann-Whitney Test. Calculations for correlations between the identified factors were also performed. A significance level of *P* < 0.05 was considered statistically significant.

## 3. Results

**Figure 1.**
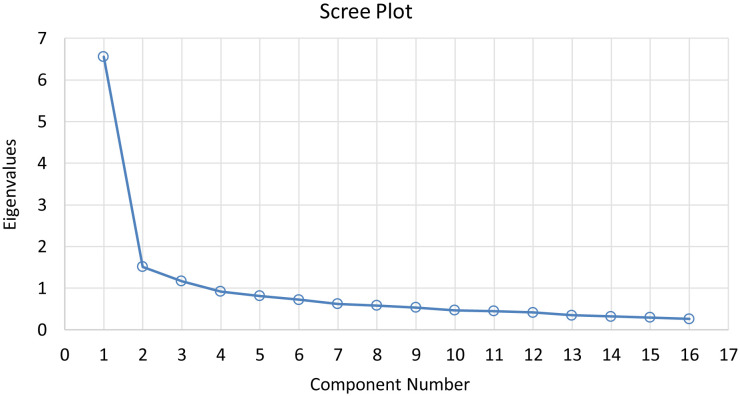
The screen plot.

A total of 382 pharmacy workers completed questionnaires. The data from the wellbeing scale in this study were suitable for factor analysis as indicated by the Kaiser–Meyer–Olkin (KMO) test resulting in a value of 0.914 and Bartlett's Test of Sphericity a p-value < 0.001. The factor extraction revealed four factors with eigenvalues greater than 1. However, in the rotated component matrix, items 10 and 14 were excluded due to each having loadings >0.4 on two factors. After re-running the factor analysis, three factors were retained as they had eigenvalues greater than 1, as indicated by the Scree plot in Figure 1. The rotated component matrix showed that each item had a single high loading >0.4 on only one factor each. This three-factor solution was deemed appropriate for our data and met the requirements of factor analysis. The total variance explained was 57.74%, and each factor comprised at least three items. The corrected item-total correlations for items within each factor were >0.3 (Factor 1: 0.45–0.67; Factor 2: 0.40–0.69; Factor 3: 0.56–0.66). The factors identified are presented in Table 1, and are as follows; Factor 1: Perceived self-worth and job satisfaction (7 items); Factor 2: Positive emotion with work (5 items); and Factor 3: Negative emotion with work (4 items).

Associations between the identified factors are presented in Table 2. Factor 1, representing perceived self-worth and job satisfaction, showed a positive correlation with Factor 2, which is indicative of positive emotions with work, with a correlation coefficient (ρ) of 0.509 (*P* < 0.01), while both Factor 1 (ρ = −0.399, *P* < 0.01) and Factor 2 (ρ = −0.416, *P* < 0.01) demonstrated negative correlations with Factor 3 (negative emotions at work). The results in Table 3 indicate that individuals with a monthly income greater than 5 million Vietnamese Dong (VND; ~$211 USD) tended to have higher perceived self-worth and job satisfaction compared to those with lower incomes (*P* = 0.016). Individuals with a monthly income greater than 5 million VND (*P* = 0.012) and those working fewer than 8 hours per day (*P* = 0.007) tended to have more positive emotions towards their work compared to others. Those aged ≤30 years (p = 0.010), those who were in the single/other marital status group (*P* = 0.014), those with a monthly income <5 million VND (*P* < 0.001), individuals working in a pharmacy chain (*P* = 0.006), and those with less than 5 years of work experience in the profession (*P* = 0.007) tended to have higher negative emotions compared to others.

**Table 1.** Rotated component matrix of the workplace wellbeing scale among pharmacy workers (n = 382).

Item	Description	Factor		
		1	2	3
	*Factor 1 – Perceived self-worth and job satisfaction*			
17	I am satisfied with my position in the organization	**0.75**	0.08	−0.32
15	I feel valued and respected by my colleagues	**0.75**	0.30	0.01
18	I feel that my contributions are recognized by my superiors	**0.73**	0.13	−0.29
11	I am satisfied with the relationships with most of my colleagues in the organization	**0.68**	0.38	0.09
16	I feel that I can demonstrate my capabilities in my work	**0.59**	0.33	−0.28
13	I feel that the outcomes of my work at the workplace are valuable	**0.56**	0.28	−0.30
12	I feel that my job is meaningful	**0.41**	0.22	−0.31
	*Factor 2 – Positive emotion with work*			
3	My work makes me feel joyful	0.27	**0.77**	−0.11
1	My work makes me feel enthusiastic	0.17	**0.76**	−0.28
5	My work makes me feel satisfied	0.23	**0.76**	−0.26
7	My work gives me motivation	0.24	**0.60**	−0.33
9	I feel that I am doing meaningful work in the organization	0.26	**0.49**	−0.12
	*Factor 3 – Negative emotion with work*			
4	My work makes me feel frustrated	−0.23	−0.12	**0.76**
2	My work makes me feel disappointed	−0.19	−0.23	**0.75**
8	My work makes me feel annoyed	−0.10	−0.15	**0.75**
6	My work makes me feel bored	−0.13	−0.34	**0.65**
	*Eigenvalue*	*6.56*	*1.52*	*1.17*
	*% of Variance*	*20.89*	*19.01*	*17.84*

Note: Extraction Method: Principal Component Analysis; Rotation Method: Varimax. Bold entries indicate the variable that has the highest factor loading on the associated factor.

**Table 2.** Associations of the identified factors in pharmacy workers (n = 382).

	Factor 1	Factor 2	Factor 3
Factor 1: Perceived self-worth and job satisfaction (7 items)	0.844^#^	0.509*	−0.399*
Factor 2: Positive emotions with work (5 items)	-	0.813^#^	−0.416*
Factor 3: Negative emotions with work (4 items)	-	-	0.795^#^

Note: *Spearman's rho indicates that the correlation was significant at the 0.01 level; ^#^Cronbach's alpha.

**Table 3.** Mean rank differences in workplace wellbeing among groups of pharmacy workers' demographic characteristics (n = 382).

Characteristics	n (%)	Perceived self-worth and job satisfaction (Mean Rank)	Variance	*P*-value	Positive emotion with work (Mean Rank)	Variance	*P*-value	Negative emotion with work (Mean Rank)	Variance	*P*-value
Age group										
≤30	320 (83.8)	189.06	0.49	0.324	189.25	0.46	0.362	197.84	0.51	**0.010**
≥31	62 (16.2)	204.11	0.35		203.11	0.36		158.76	0.37	
Gender										
Woman	304 (79.6)	192.97	0.48	0.607	192.72	0.46	0.667	194.04	0.48	0.369
Man	78 (20.4)	185.78	0.42		186.73	0.4		181.59	0.55	
Marital status										
Single/other	277 (72.5)	189.86	0.52	0.636	188.35	0.49	0.362	199.94	0.47	**0.014**
Married	105 (27.5)	195.83	0.33		199.80	0.32		169.23	0.56	
Education^†^										
Intermediate/college	160 (41.9)	195.41	0.43	0.556	201.96	0.47	0.113	185.65	0.37	0.374
University/postgraduate	222 (58.1)	188.68	0.5		183.96	0.42		195.72	0.58	
Monthly income										
<5 million VND	191 (50)	177.89	0.46	**0.016**	177.45	0.42	**0.012**	212.30	0.54	**<0.001**
≥5 million VND	191 (50)	205.11	0.47		205.55	0.46		170.70	0.42	
Type of pharmacy										
Independent pharmacy	215 (56.3)	195.71	0.41	0.396	190.49	0.39	0.839	177.96	0.46	**0.006**
Pharmacy chain	167 (43.7)	186.08	0.54		192.80	0.52		208.94	0.52	
Job position										
Pharmacy staff	288 (75.4)	191.57	0.52	0.983	194.95	0.5	0.282	193.90	0.51	0.451
Pharmacy manager	94 (24.6)	191.29	0.32		180.94	0.27		184.13	0.45	
Duration of work at the pharmacy		
<3 years	316 (82.7)	192.91	0.48	0.583	189.29	0.47	0.389	195.51	0.49	0.116
≥3 years	66 (17.3)	184.73	0.44		202.08	0.32		172.30	0.49	
Work experience in the profession		
<5 years	299 (78.3)	188.46	0.5	0.307	189.39	0.49	0.476	199.43	0.51	**0.007**
≥5 years	83 (21.7)	202.43	0.34		199.08	0.28		162.93	0.42	
Working hours per day										
<8 hours	44 (11.5)	208.30	0.46	0.282	233.34	0.34	**0.007**	174.84	0.36	0.282
≥8 hours	338 (88.5)	189.31	0.47		186.05	0.45		193.67	0.51	

Note: The Mann-Whitney U test was used. Bold entries indicate statistical significance. ^†^Intermediate/college level refers to intermediate or college-level pharmacy workers with basic knowledge and skills, graduating from a 2–3 years program, while University/postgraduate level refers to undergraduate pharmacy workers (graduating from a 5-year program) and postgraduate pharmacy workers (graduating from a program of 2 or more years) with deeper knowledge and the ability to engage in research and drug development.

## 4. Discussion

Wellbeing is a term that reflects not only an individual's health but also their satisfaction with work and life [22]. The nature of work can be both positive and negative, and workplace wellbeing can significantly impact employees' behaviors and performance. Wellbeing is crucial in all professions, but particularly for healthcare professionals [22]. As the work of a pharmacist involves direct contact with customers/patients, they need to be confident, creative, compassionate, and enthusiastic, as all these characteristics not only ensure the delivery of good patient care but are directly modified by an employee's wellbeing [23]. Using factor analysis, we found the workplace wellbeing of pharmacy workers to comprise three fundamental aspects: Factor 1 – perceived self-worth and job satisfaction, Factor 2 – negative emotions in the workplace, and Factor 3 – positive emotions in the workplace.

### 4.1. Correlation between identified factors

We found a positive correlation between perceived self-worth, job satisfaction, and positive emotions in the workplace. This finding is consistent with the notion that self-esteem, defined as a person's sense of self-worth [24], is closely associated with positive emotions. Self-esteem allows an individual to feel comfortable with themselves, reinforcing or prolonging positive emotions [25],[26]. High self-esteem leads an individual to experience more positive emotions, characterized by vigor, strength, stability, and conscientiousness [27]. Furthermore, previous research findings support the argument that job satisfaction was strongly influenced by felt emotions. Therefore, the higher the emotional regulation, the greater the job satisfaction [28]. We found similar results to Kammeyer-Mueller et al. (2013), which concluded that positive emotions were strongly correlated with the perception of high job satisfaction, while negative emotions were correlated with the perception of low job satisfaction [29]. Therefore, creating a work environment that emphasizes emotional goals alongside financial goals will help provide a positive work environment, facilitate the use of strategies to regulate emotions, and lead to higher levels of compliance with employee expectations, in turn resulting in higher job satisfaction. Gander et al. discovered that workplace activities designed to elicit positive experiences such as joy, meaning, and accomplishment were effective in promoting subjective and physical wellbeing in the workplace [30]. Therefore, incorporating such activities into the workplace can contribute to overall employee wellbeing.

### 4.2. Differences in workplace wellbeing across demographic groups

Workplace wellbeing, productivity, and population wellbeing are influenced by various factors. According to Schulte and Vainio, workforce wellbeing was influenced by workplace factors, socioeconomic status, host and demographic factors, health, occupational hazards, and environmental factors [22]. We identified several factors that influence the workplace wellbeing of pharmacy workers. Age group and work experience in the profession were found to be associated with negative emotions at work. The daily tasks of a community pharmacist involve multiple responsibilities such as communication, counseling, dispensing medications to consumers/patients, and ensuring medication safety through sorting, storage, preparation, expiration date checking, and updating medication information [10]. To be able to perform these tasks effectively, pharmacists undergo training, internships, and develop various skills such as communication skills, counseling skills, stress tolerance, and patience [31]. Therefore, older experienced pharmacists have better emotional regulation and coping strategies in the workplace. Numerous studies have demonstrated associations between age and emotional regulation [32]. As workers age, their emotions tend to improve, and older workers may maintain better job-related happiness compared to younger workers. Positive changes in emotional experiences, emotion expression, and emotional competencies appear in the later stages of a career [32]. It is necessary to develop training courses for pharmacy workers that provide knowledge, skills, and psychological training for younger employees, create a positive work environment, set goals, and provide corresponding rewards upon task completion as solutions that pharmacy managers can implement to improve emotional wellbeing.

Work and family are crucial aspects of life, providing meaning and identity to individuals as they balance their commitments and affiliations with social structures. Balancing the roles of work and family is a daily challenge for millions of adults, and has been so for decades [33]. We revealed a significant association between marital status and negative emotions in the workplace, which is consistent with previous research. Specifically, individuals with a marital status of single/other exhibited higher levels of negative emotions compared to married individuals, which agrees with the findings of Hsu and Barrett (2020) [34],[35]. A recent study reported significantly higher wellbeing scores among married pharmacy students [2],[34],[36]. Marriage can reduce vulnerability to psychological disorders and bring health benefits, contributing to personal and economic development. Pharmacists face high workloads and professional stress, highlighting the need for clear work strategies to ensure a healthy work-life balance for employees. Furthermore, seeking input from support groups and family members can help alleviate stress.

We also found a significant difference in self-worth and job satisfaction associated with income, consistent with previous research [14],[37],[38]. Economic challenges can impact pharmacy profitability, leading to staff increasing their working hours to maintain the same level of income. This can decrease job satisfaction, increase psychological stress, and in turn affect productivity and personal wellbeing [39]. Pharmacists who are satisfied with their work are more motivated to provide better patient care [38]. Therefore, improving income for pharmacists can enhance job satisfaction and workplace wellbeing.

In this study, workers employed in pharmacy chains were found to experience significantly higher levels of negative emotions compared to their counterparts in independent pharmacies. The pharmaceutical market in Vietnam has undergone significant changes in recent years, with the emergence of prominent domestic and international brands and an increasing number of pharmacy chains competing with independent pharmacies [40]. In addition to fundamental skills, pharmacy workers in pharmacy chains are required to acquire additional skills to enhance their competitive edge, including the use of modern technology and the intensification of advertising and promotions to attract customers. The pressures arising from this competition may contribute to the higher levels of negative emotions observed in pharmacy chains in our study.

Pharmacy workers surveyed who worked less than 8 hours per day were found to have significantly higher levels of positive emotions compared to those working 8 hours or more. Long working hours increases stress and reduces the quality of life of employees [39]. Studies on shift length in healthcare workers have shown that 3.4% of those working 12-hour shifts and 3% of those working 8-hour shifts had a diagnosis of severe depression [41]. Pharmacists in community pharmacies should have autonomy over their working hours to reduce stress and increase levels of positive emotions [12].

Pharmaceutical care plays a role in reducing the incidence and mortality associated with medication-related issues, improving clinical outcomes and health-related quality of life, and reducing healthcare costs [42]. The foundation of success in pharmaceutical care lies with the pharmacy worker. Therefore, leadership and management in the workplace play crucial roles in creating a positive work environment and promoting wellbeing. Additionally, managers, policymakers, and stakeholders in the healthcare industry should prioritize the health, mental wellbeing, and welfare of pharmacy workers, and pharmacy workers themselves should have access to healthcare services to enhance their workplace wellbeing.

### 4.3. Recommendations for future studies

Qualitative studies in the future could be beneficial in identifying the causes leading to workplace wellbeing in pharmacy workers. Additionally, the relationship between workplace wellbeing and other factors such as burnout, stress, anxiety, depression, etc., should also be evaluated to find better intervention solutions. The results of this study could serve as a foundation for expanding the scope to other healthcare professionals, such as doctors and nurses, in Vietnam.

### 4.4. Study Limitations

This study has several limitations. First, the study sample was selected from pharmacies in only 4 districts in Can Tho City, Vietnam. Therefore, the results may not be generalizable to all pharmacies in Can Tho. The use of Google Maps as a pharmacy locator may have resulted in the exclusion of some very new or non-tech-savvy pharmacy locations that were not listed on Google Maps. Consequently, these locations did not have the opportunity to participate in our study. Second, the data collection relied on self-administered questionnaires, which may introduce risks of bias through inaccurate or dishonest responses from participants. Third, we did not include any longitudinal follow-up as the design was cross-sectional, meaning that data was only collected at a single time point. This does not allow any assessment of changes in wellbeing over time. Last, in measuring wellbeing, the study primarily focused on the aspects of self-worth, job satisfaction, and positive and negative emotions. However, wellbeing encompasses multiple other dimensions that were not covered. These limitations should be considered and addressed in the design of future studies to ensure a more comprehensive understanding of wellbeing in the community pharmacy setting.

## 5. Conclusion

Our results revealed three fundamental aspects of workplace wellbeing: Perceived self-worth and job satisfaction, positive emotions with work, and negative emotions with work. Perceived self-worth and job satisfaction exhibited a positive correlation with positive emotions at work, while perceived self-worth, job satisfaction, and positive emotions at work demonstrated a negative correlation with negative emotions at work. Additionally, higher income was associated with higher positive emotions with work, higher perceived self-worth and job satisfaction, and lower negative emotions with work. Our findings suggest that leaders, managers in the workplace, as well as policymakers, should implement policies aimed at improving income for pharmacy workers, which could lead to benefits in enhancing job satisfaction and workplace wellbeing.

## Use of AI tools declaration

The authors declare they have not used Artificial Intelligence (AI) tools in the creation of this article.


