# Supplementary material for: Workplace wellbeing in community pharmacy practice: A cross-sectional study in Can Tho, Vietnam
Source: AIMS Public Health. 2024 Mar 11;11(1):258–72. doi: 10.3934/publichealth.2024013 (PMC11007423; doi:10.3934/publichealth.2024013)
Supplement: Supplementary file 1 [file publichealth-11-01-013-s001.pdf]

Public Health

AIMS Public Health, 11(1): 258–272.
DOI: 10.3934/publichealth.2024013
Received: 07 January 2024
Revised: 22 February 2024
Accepted: 28 February 2024
Published: 11 March 2024

http://www.aimspress.com/journal/aimsph

Research article

Workplace wellbeing in community pharmacy practice: A cross-sectional study in Can Tho, Vietnam

Van De Tran[1], Trung Tin Pham[1], Trung Hieu Le[1], Thanh Thao Nguyen Thi[1], Minh Trung Nguyen[2], Duong Phuc Phan[3], Thi Bich Thuy Bui[4], Minh Cuong Nguyen[5], Rebecca Susan Dewey[6] and Nguyet Tu Tran[7,*]

[1] Department of Health Organization and Management, Can Tho University of Medicine and Pharmacy, 179 Nguyen Van Cu Street, Can Tho 900000, Vietnam
[2] Department of Epidemiology, Can Tho University of Medicine and Pharmacy, 179 Nguyen Van Cu Street, Can Tho 900000, Vietnam
[3] Department of Nutrition and Food Safety, Can Tho University of Medicine and Pharmacy, 179 Nguyen Van Cu Street, Can Tho 900000, Vietnam
[4] Administration Office, Faculty of Public Health, Can Tho University of Medicine and Pharmacy, 179 Nguyen Van Cu Street, Can Tho 900000, Vietnam
[5] Faculty of Pharmacy, Nam Can Tho University, 168 Nguyen Van Cu Street, Can Tho 900000, Vietnam
[6] Sir Peter Mansfield Imaging Centre, School of Physics and Astronomy, University of Nottingham, Nottingham NG7 2RD, United Kingdom
[7] Department of Environmental Health, Can Tho University of Medicine and Pharmacy, 179 Nguyen Van Cu Street, Can Tho 900000, Vietnam

* **Correspondence:** Email: ttnguyet@ctump.edu.vn; Tel: +84774023152; Fax: +842923740221.

Supplementary

Questionnaire: Workplace wellbeing in community pharmacy practice: a cross-sectional study in Can Tho, Vietnam.

A. Participant information

Age (year):...

Gender:

□ Woman

□ Man

Marital Status:

□ Single

□ Married

□ Other

Pharmacy educational Level:

□ Intermediate degree

□ College degree

□ University degree

□ Master's degree/Specialist degree I

□ Doctorate degree/Specialist degree II

Average monthly income (in million VND):...

Type of pharmacy

□ Independent pharmacy

□ Pharmacy chain

Job position

□ Pharmacy staff

□ Pharmacy manager

Duration of work at the pharmacy (year):...

Work experience in the profession (year): ....

Working hours per day (hour): ....

B. Assessment of workplace wellbeing

1-Strongly Disagree; 2-Disagree; 3-Neutral; 4-Agree; 5-Strongly Disagree

Item	Description	1	2	3	4	5
1	My work makes me feel enthusiastic	□	□	□	□	□
2	My work makes me feel disappointed	□	□	□	□	□
3	My work makes me feel joyful	□	□	□	□	□
4	My work makes me feel frustrated	□	□	□	□	□
5	My work makes me feel satisfied	□	□	□	□	□
6	My work makes me feel bored	□	□	□	□	□
7	My work gives me motivation	□	□	□	□	□
8	My work makes me feel annoyed	□	□	□	□	□
9	I feel that I am doing meaningful work in the organization	□	□	□	□	□
10	I am satisfied with the income from my job at the organization	□	□	□	□	□
11	I am satisfied with the relationships with most of my colleagues in the organization	□	□	□	□	□
12	I feel that my job is meaningful	□	□	□	□	□
13	I feel that the outcomes of my work at the workplace are valuable	□	□	□	□	□
14	I am satisfied with the job I currently have	□	□	□	□	□
15	I feel valued and respected by my colleagues	□	□	□	□	□
16	I feel that I can demonstrate my capabilities in my work	□	□	□	□	□
17	I am satisfied with my position in the organization	□	□	□	□	□
18	I feel that my contributions are recognized by my superiors	□	□	□	□	□

AIMS Press

© 2024 the Author(s), licensee AIMS Press. This is an open access article distributed under the terms of the Creative Commons Attribution License (http://creativecommons.org/licenses/by/4.0).
